# Finsen Light

**Published:** 1902-09-27

**Authors:** 


### Editor's Letter-Box.

[Our Correspondents are reminded that prolixity is a great bar to publication, and that brevity of style and conciseness of statement greatly facilitate early insertion.]

### FINSEN LIGHT.

F. Clare Melhado, Secretary-Superintendent, the Middlesex Hospital, writes:—"In a paragraph under 'Notes and News' in your issue dated 20th instant, you refer to those hospitals which use the Finsen Light. I thought you might be interested to hear that this hospital is just putting up a temporary building in the courtyard, which is to be fitted up with the Finsen Light, X-Rays and other electrical appliances."

C. B. Keetley, 56 Grosvenor Street, W., writes:—

In your issue of August 30th, p. 377, it is reported that Dr. Robert F. Weir, of New York, has shown that the "otherwise useless appendix" vermiformis "can be put to some useful purpose," and you then describe how, instead of doing a right inguinal colostomy or a cæcostomy, he brought the appendix into the parietal wound and, opening it, used it as a mode of access to the inside of the cæcum.

The possibility of, and the advantages of this particular method were pointed out by me at the Medical Society of London on November 12th, 1894, *i.e.* eight years ago. There is, not a report of, but a brief reference to my remarks on the subject in the Society's Transactions, vol. xviii. p. 361, as well as in the *British Medical Journal* and in the *Lancet* of November 17th.

An idea so simple and natural as this no doubt occurred to Dr. Weir independently. Indeed, my remarks seem to have attracted little or no attention, probably because the degree to which a normal appendix can be dilated is not generally known. As it is likely that not a few normal appendices are now being removed, knowledge is no doubt advancing on this point.

